# Order of arrival and nutrient supply alter outcomes of co-infection with two fungal pathogens

**DOI:** 10.1098/rspb.2024.0915

**Published:** 2024-08-28

**Authors:** Elizabeth T. Green, Rita L. Grunberg, Charles E. Mitchell

**Affiliations:** ^1^ Department of Biology, University of North Carolina at Chapel Hill, Chapel Hill, NC, USA; ^2^ School of Plant Sciences, University of Arizona, Tucson, AZ, USA; ^3^ Wilson Center for Science and Justice, Duke University, Durham, NC, USA

**Keywords:** priority effects, growth-defence trade-offs, disease ecology, plant disease, community ecology

## Abstract

A pathogen arriving on a host typically encounters a diverse community of microbes that can shape priority effects, other within-host interactions and infection outcomes. In plants, environmental nutrients can drive trade-offs between host growth and defence and can mediate interactions between co-infecting pathogens. Nutrients may thus alter the outcome of pathogen priority effects for the host, but this possibility has received little experimental investigation. To disentangle the relationship between nutrient availability and co-infection dynamics, we factorially manipulated the nutrient availability and order of arrival of two foliar fungal pathogens (*Rhizoctonia solani* and *Colletotrichum cereale*) on the grass tall fescue (*Lolium arundinaceum*) and tracked disease outcomes. Nutrient addition did not influence infection rates, infection severity or plant biomass. *Colletotrichum cereale* facilitated *R. solani*, increasing its infection rate regardless of their order of inoculation. Additionally, simultaneous and *C. cereale*-first inoculations decreased plant growth and—in plants that did not receive nutrient addition—increased leaf nitrogen concentrations compared to uninoculated plants. These effects were partially, but not completely, explained by the duration and severity of pathogen infections. This study highlights the importance of understanding the intricate associations between the order of pathogen arrival, host nutrient availability and host defence to better predict infection outcomes.

## 1. Introduction

A pathogen arriving on a host typically encounters a diverse community of symbionts and other pathogens that have previously colonized that host [1]. The later-arriving pathogen thus enters a within-host environment that may have been shaped by earlier-arriving species in multiple ways, including the usage of nutrients and other resources [2] and modulation of host immune or defence responses [3]. The effects that an earlier-arriving species has on the success of a later-arriving species, as a consequence of their difference in arrival time, are called priority effects [4–6]. Within-host priority effects can be either inhibitive [7], when an earlier-arriving species makes the host less habitable for later-arriving species, or facilitative [8], when an earlier-arriving species makes it easier for a later-arriving species to establish, reproduce or grow [9–11]. By influencing the pathogens’ ecological interactions within the host, the order in which pathogens arrive on a host can shift the infection outcomes for not only the pathogens but also the host [4,8,12]. As these within-host priority effects can result in different epidemic trajectories and require adaptable interventions [7,11,13–15], it is important to understand how within-host priority effects are influenced by within-host conditions and resources.

To grow or replicate within a host, pathogens must acquire nutrients and other resources. Earlier-arriving pathogens can deplete the availability of within-host resources, potentially reducing the infection success of later-arriving pathogens [2,16]. Once a pathogen has successfully infected the host, its growth or replication and thus the severity of its infection can depend on competition from other pathogens and the resource availability in the host tissue [17,18]. Increasing within-host resource availability can increase pathogen infection success, replication or growth [2] suggesting that resource addition could influence within-host pathogen priority effects [1], but this possibility has received little experimental investigation.

As well as competition for resources, priority effects can also be mediated by host defences [3,12,19–21]. Different types of pathogens can trigger different plant defence pathways [22–24]. There is negative crosstalk between many of these defence pathways, wherein a host cannot simultaneously induce two pathways, leaving the plant susceptible to infection by one of the attacking pathogens [3,24,25]. In this context, two important types of pathogens are necrotrophs, which kill leaf tissue and extract nutrients from the dead or dying tissue, and biotrophs, which extract nutrients from living leaf tissue. When a necrotrophic pathogen infects a plant host, it triggers the jasmonic acid (JA) signalling pathway [26], while attack by a biotrophic pathogen triggers the salicylic acid (SA) pathway [23]. The negative crosstalk between the two immune pathways [20,24] may mediate facilitative priority effects between co-infecting pathogens [23,27].

The availability of nutrients to pathogens within a host can be driven by the environmental nutrient availability [14,28,29], and environmental nutrient supplies can also influence host–pathogen interactions by changing host growth and defence strategies [22,30,31]. Both growth and defence require resources, so host allocation to defence often comes at the expense of growth and vice versa. Whether a host allocates resources towards growth or defence is dependent on the resources available to it [28,30]. While growth-defence trade-offs have been well studied in many herbivores and some pathogen systems [3,22], the implications for pathogen co-infection are largely unknown.

Despite the potential for nutrient availability to influence both the priority effects of pathogens within hosts and host allocation to growth and defence, there has been little experimental work to investigate how infection outcomes are shaped by interactions between order of arrival and nutrient availability [32,33]. As a step to fill this gap, we used an experimentally tractable system, the grass species tall fescue (*Lolium arundinaceum*) and two fungal pathogens, *Rhizoctonia solani* and *Colletotrichum cereale. Rhizoctonia solani* and *C. cereale* commonly co-infect tall fescue in the field [15,34–36]. Furthermore, they have the potential to interact within a host, both by competition for resources such as nutrients, and by inducing negative crosstalk between the JA and SA defence pathways—a mechanism of facilitation. Indeed, *C. cereale* has previously been found to facilitate infection by *R. solani* [11,15]. Here, we conducted an experiment that factorially manipulated the order of arrival of *R. solani* and *C. cereale* on the plant, and the nutrient availability to the plant, for the purpose of testing three predictions about infection rates and outcomes. First, we predicted that the order of pathogen arrival and nutrient availability will affect infection rates by modulating host susceptibility and defence against infection. Second, we predicted that nutrient availability and pathogen order of arrival will influence the severity of infections by mediating interactions between co-infecting pathogens. Finally, we predicted that co-infection will reduce host growth more under low-nutrient conditions, owing in part to resources spent on plant defence and a trade-off between growth and defence. Testing these three predictions will help to understand the dynamics and outcomes of co-infection under differing environmental nutrient scenarios as humans increasingly alter environmental nutrient supplies [14].

## 2. Methods

### (a) Study system

Tall fescue (*L. arundinaceum*) is a common grass species across the southern and eastern United States [36,37]. Tall fescue is host to numerous fungal pathogens, including those infecting both the roots and the leaves. The widespread abundance of tall fescue, its sensitivity to nutrients and its rich community of pathogens make it an ideal system to study ecological interactions between nutrients, host, and pathogens [8,15,36]. In this study, we focus on two fungal pathogens that frequently co-infect leaves but occupy different trophic niches. *Rhizoctonia solani* is a necrotroph. Upon infection, it kills leaf tissue causing necrotic lesions and can ultimately kill the whole leaf. It is soil and water borne and spreads through asexual hyphal growth and causes the disease brown patch, locally killing clusters of plants [38]. *Colletotrichum cereale* is one of several species responsible for anthracnose disease in tall fescue [39]. It is a hemibiotroph, initially infecting and extracting nutrients from living cells as a biotroph, then changing its feeding strategy to kill leaf tissue causing necrotic lesions. Splashing rain drops disperse asexual condiospores between leaves. Recent studies have found that on tall fescue in the Piedmont region of North Carolina USA, *C. cereale* arrives on leaves starting in April and *R. solani* follows in late June [40]. At the peak of the seasonal epidemics, 80% of surveyed leaves are infected with *C. cereale*, while up to 30% of leaves experience *R. solani* infection [35]. Many of those surveyed leaves exhibit lesions from both pathogens, up to 11% of leaves at the peak of the epidemics (E.T. Green 2023, unpublished data). In a growth chamber experiment in tall fescue, *C. cereale* has been found to facilitate growth of *R. solani* when arriving first, leading to an increased disease severity [11].

### (b) Experimental overview

The fungal cultures and seed used in this experiment were collected from Widener Farm, an old field that was abandoned from agriculture in 1996, has been mown at least annually since then, and is part of Duke Forest Research and Teaching Laboratory, Orange County, NC, USA (36.008891° N–79.018263° W). The *R. solani* and *C. cereale* cultures were both collected in 2015 by F. Halliday and K. O’Keeffe respectively. This experiment used our *C. cereale* isolate coded 6B and our *R. solani* isolate coded S1W6, the same isolates used in [11,11] and Grunberg *et al.* [34]. Tall fescue is commonly infected by the vertically transmitted fungal endophyte *Epichloë coenophiala*, which can increase the plant’s drought and heat stress tolerance and provide resistance to herbivory [41–45]. The tall fescue seed in this experiment was part of an accession collected in 2018 by B. Joyner. In a sample from this accession, over 90% of the seeds tested positive for *Epichloë* (as tested B. Joyner in 2018).

We ran the experiment twice in 2022. For the first temporal replicate, we germinated plants on 28 February and harvested them on 9 May and for the second temporal replicate, we germinated plants on 11 April and harvested them on 15 June (electronic supplementary material, table S1). The first replicate was 5 days longer from germination to harvest. We applied two treatments to each plant: a low (50 ppm) or high (150 ppm) fertilizer treatment (20N–20P–20K Peter’s Original Water Soluble Fertilizer) and a fungal inoculation treatment (table 1). Nutrient addition treatments were chosen to scale parts per million from field populations, where previous studies have shown effects on plant biomass and *R. solani* infection, to individual plants [36]. In each replicate, the plants were randomly assigned a treatment group and the treatments were factorially applied. We grew the plants in a greenhouse until they were inoculated and then moved them to either two growth chambers (replicate 1) or a grow room (replicate 2). The spatial (two growth chambers) and temporal replicates were represented as three blocks in all further analyses. Both locations were kept at 29°C with constant light. While we initially assigned 44 plants to each inoculation group (30 plants in the mock inoculation group), *C. cereale* spore production is highly variable, so we ran out of *C. cereale* spores and did not have enough inoculum to complete the original plan in the first replicate. To adjust, we substituted an *R. solani* inoculation in place of *C. cereale* for some of the plants that were originally assigned a *C. cereale* inoculation in the first replicate and reduced the number of plants in *R. solani* inoculation groups in the second replicate. This resulted in some treatment groups having a different number of inoculated plants than originally planned. In total, there were 250 plants, six of which tested negative for the vertically transmitted endophyte *E. coenophiala* and were removed from the data analysis (for treatment replication see table 2).

**Table 1.** Inoculation treatments.

inoculation	day 1	day 2
mock	mock *C. cereale* and mock *R. solani*	NA
*R. solani* only	*R. solani*	mock *C. cereale*
*C. cereale* only	*C. cereale*	mock *R. solani*
*R. solani* first	*R. solani*	*C. cereale*
*C. cereale* first	*C. cereale*	*R. solani*
simultaneous	*C. cereale* and *R. solani*	NA

**Table 2.** Number of plants per inoculation group. (From 250 plants total, six *E. coenophiala-*negative plants were excluded and 244 *E. coenophiala*-positive plants were retained for analyses.)

inoculation	planned replicate 1	replicate 1	planned replicate 2	replicate 2	total *E. coenophiala* positive plants
mock	20	20	10	10	30
*R. solani* only	24	31 (one *E. coenophiala* negative)	20	15	45
*C. cereale* only	24	18 (one *E. coenophiala* negative)	20	20	37
*R. solani* first	24	29 (one *E. coenophiala* negative)	20	15 (one *E. coenophiala* negative)	42
*C. cereale* first	24	22	20	20 (one *E. coenophiala* negative)	41
simultaneous	24	24	20	20 (one *E. coenophiala* negative)	43

### (c) Experimental set-up

Seeds were primed by soaking in water for 6 h and allowed to dry overnight. The seeds were sprinkled over moist vermiculate and left in the greenhouse with a 12 L : 12 D cycle to germinate for 14 days. We haphazardly selected seedlings to transplant one seedling into each 102 cm^3^ deepot packed with MetroMix 360 potting soil and left them to grow for another two weeks in the greenhouse. Plants were watered twice per week throughout the experiment before applying the fertilizer mixture to reduce run off. We then started manually applying 10 ml of the assigned fertilizer concentration (low or high) mixed in water twice per week.

*Rhizoctonia solani* was grown on potato dextrose agar (PDA) and frozen in potato dextrose broth at −80°C. *Colletotrichum cereale* was grown on PDA slants and stored at 4°C. Three weeks before inoculation, five plugs of *R. solani* hyphae were pulled from the freezer and plated on plates of PDA. Cultures were left to grow at 25°C with constant light for 7 days and then replated to multiply the number of active cultures.

At the same time, five *C. cereale* plugs were pulled from PDA slants in storage and plated on fresh PDA plates. Plates were left to grow for two weeks at 25°C under constant light. We then flooded the *C. cereale* plates with 8 ml of water and scraped them with a cell scraper to loosen the spores. The spore solution was collected and 0.5 ml of solution was spread on each new plate. The plates were left unparafilmed to stress the cultures and encourage sporulation. After 5 days, each *C. cereale* plate was flooded again with 10 ml of sterile water and scraped to release spores. The spore slurry was collected and the spore density from each plate was estimated by looking at the slurry on a haemocytometer under a microscope. The combined spore concentrations were 690 000 conidiospores ml^−1^ in the first repetition and 568 200 conidiospores ml^−1^ in the second.

### (d) Inoculations

We randomly assigned plants to one of the six experimental treatments with an equal number between fertilizer treatments (mock *C. cereale* and mock *R. solani,* only *C. cereale*, only *R. solani*, *C. cereale* followed by *R. solani, R. solani* followed by *C. cereale*, and both *R. solani* and *C. cereale* simultaneously; table 1). The plants that only received one inoculation were treated with a mock version of the other inoculation (potato dextrose broth or a plug of PDA) on the second inoculation day. We haphazardly selected a tiller, shoot growing from the base of the plant, from one to five tillers of each plant and inoculated the second youngest leaf of that tiller. The tiller was stabilized with a wooden stake and marked by wrapping a single plastic comb binding around the tiller. We first painted the leaf with 0.5 ml of the *C. cereale* spore solution or mock potato dextrose broth solution, if receiving that treatment. We then gently wrapped the leaf in tinfoil at the base of the leaf, leaving a gap between the tiller and leaf. With a cork borer sterilized with ethanol and run through flame, we pulled a plug from the outer edge of either active *R. solani* culture or sterile PDA plate and nestled the plug between the leaf and tiller. A piece of cotton was soaked in sterile water and placed on top of the plug before closing the tinfoil wrap around both the plug and cotton. We finished each plant by wrapping the tinfoil casing with parafilm to keep in the moisture. Finally, plants were placed in dew chambers made of plastic bags spritzed with water and rubber banded at the base of the pot. Plants were moved to growth chambers or a grow room with constant light and temperature of 29°C and placed in tubs of water to bottom water.

After 48 h, we removed the plastic bag, parafilm, tinfoil, cotton, fungal plug and stake. Four days after the first round of inoculations, we completed the second round of inoculations for the plants in the co-inoculation treatments or the mock treatment for those receiving a single inoculation.

### (e) Infection surveys

We surveyed the plants five times, every 2–3 days, in each replicate (electronic supplementary material, table S1). The experiment was ended before lesions became severe enough to kill the inoculated leaves, 18 days after inoculation in the first replicate and 16 days after inoculation in the second replication (electronic supplementary material, table S1). We examined the inoculated leaf for visible *R. solani* and *C. cereale* lesions and measured the total length of each lesion (electronic supplementary material, figure S1).

At the end of the surveys, we collected the inoculated leaf from every plant and placed them in paper bags for elemental analysis. We also collected a 1 cm tiller sample to test for the fungal endophyte, *E. coenophiala* (Agrinostics, Phytoscreen field tiller endophyte detection kit). The prevalence of *E. coenophiala* was 97.6% in all plants, so we removed the six *Epichloë*-negative plants from the sample. The rest of the plant was collected and dried to measure aboveground biomass. The dried inoculated leaves were ground in a Retsch mixer ball mill. We weighed out between 1.0 and 3.0 mg of ground leaf tissue and packed it into tin capsules. Samples were shipped to the Stable Isotope Analysis Laboratory at the University of Georgia for elemental analysis. Leaves were analysed for total carbon (%C) and nitrogen (%N) content and their corresponding elemental ratio (C : N). For analyses of biomass and nutrients, we excluded the 117 plants that were not symptomatic for the inoculated pathogen by the end of the survey period, leaving 112 plants. We also removed the leaves that were more than 50% chlorotic from the nutrient analyses, leaving 94 plants.

### (f) Data analysis

All analyses were completed in R (4.2.1) and linear models (lm, stats) were performed with a fixed effect of experimental block and a full factorial of nutrient and inoculation treatment. We ran Tukey’s honest significant difference (HSD) as a pairwise comparison between inoculation groups and nutrients for all linear models (emmeans) [46].

We ran all infection analyses for both *R. solani* and *C. cereale*. We analysed disease progression for the first four surveys after inoculation, because the sequential inoculations only had four surveys after the second inoculation. To assess the rate of infection in each group, we performed a survival analysis of infection incidences across the survey period using a Cox proportional hazards model, a regression model of infection incidence that evaluates the effects of nutrients, inoculation group and their interaction on time to infection events [47]. Cox proportional hazard models provide both an effect estimate and confidence intervals for an infection rate [48]. To analyse infection severity independently of infection success, treatment effects were analysed separately for each disease, including only plants symptomatic for that disease (*C. cereale n* = 98, *R. solani n* = 107). For both infection types, we calculated the area under the disease progression stairs (AUDPS) of lesion length across the survey days (epifitter) [49]. AUDPS as a metric of disease severity provides an advantage over other metrics like the area under the disease progression curve because it better accounts for the contribution of the first and last survey measurements [50]. We then log-transformed the AUDPS values to minimize heteroskedasticity and performed a linear model of the AUDPS value.

To assess plant growth and nitrogen concentration, we ran three linear models with aboveground biomass, and log-transformed C : N ratio [51] and total N (%) as the dependent variable. The C : N ratio measures how nutrient-rich leaf tissue is in comparison to the structural carbon. Nitrogen is often a limiting nutrient in photosynthesis and production of proteins. A high ratio suggests a leaf that is nitrogen limited [52].

### (g) Post hoc hypothesis

After our pre-planned analyses above, the results raised the question of whether treatment effects on biomass and leaf nutrients could be explained by the duration of, or time-integrated severity of infection (AUDPS). To answer this question, we performed a post hoc analysis in which we added the duration (in days) and severity (AUDPS) of *R. solani* and *C. cereale* infection to the above models, entering those terms in the model before the factorial treatments.

## 3. Results

### (a) Rate of infection

**Figure 1.**
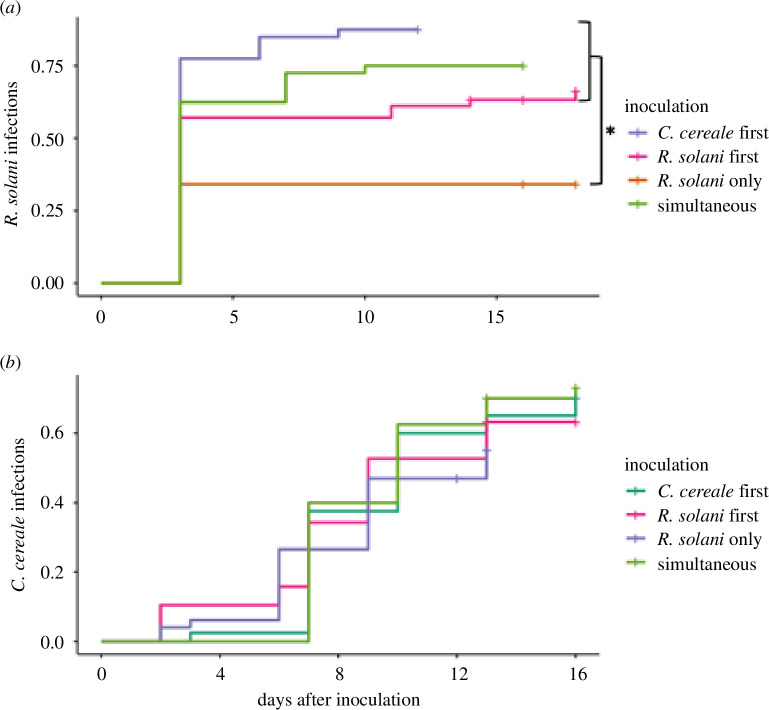
Kaplan–Meier curves of *R. solani* (*a*) and *C. cereale* (*b*) infection incidence on host tall fescue by inoculation treatment. Day 0 is the day of inoculation. Data shown are grouped across nutrient treatments (50 or 150 ppm N–P–K). In a Cox proportional hazard model, *R. solani* infection rate from day 0 through to 16 after inoculation (*a*) when inoculated alone (orange) was significantly lower than in the three treatment groups co-inoculated with *C. cereale*, as noted by the asterisk.

We conducted a survival analysis with a Cox proportional hazard model to assess infection rates between treatment groups. When plants were inoculated with only *R. solani* their infection rate with *R. solani* was 45% lower when compared to the inoculation treatment with the next lowest final infection rate (*R. solani* first*; p* = 0.008; electronic supplementary material, table S2A; figure 1*a*). Generally, *C. cereale* inoculation facilitated infection by *R. solani* regardless of replicate, nutrient treatment, or order of arrival (ANOVA; replicate: $\chi_{2,146}^{2}$ = 0.83, *p* = 0.66; inoculation: $\chi_{1,146}^{2}$ = 26.19, *p* < 0.001; nutrients: $\chi_{3,146}^{2}$ = 0.74, *p* = 0.39; inoculation × nutrients: $\chi_{3,146}^{2}$ = 1.42, *p* = 0.70; electronic supplementary material, table S2B). However, neither *R. solani* inoculation treatment nor nutrients affected the rate of plant infection by *C. cereale* (ANOVA; inoculation: $\chi_{3,141}^{2}$ = 0.88, *p* = 0.83; nutrients: $\chi_{1,141}^{2}$ = 1.51, *p* = 0.22; inoculation × nutrients: $\chi_{3,146}^{2}$ = 1.61, *p* = 0.66; electronic supplementary material, table S2; figure 1*b*).

### (b) Disease severity

**Figure 2.**
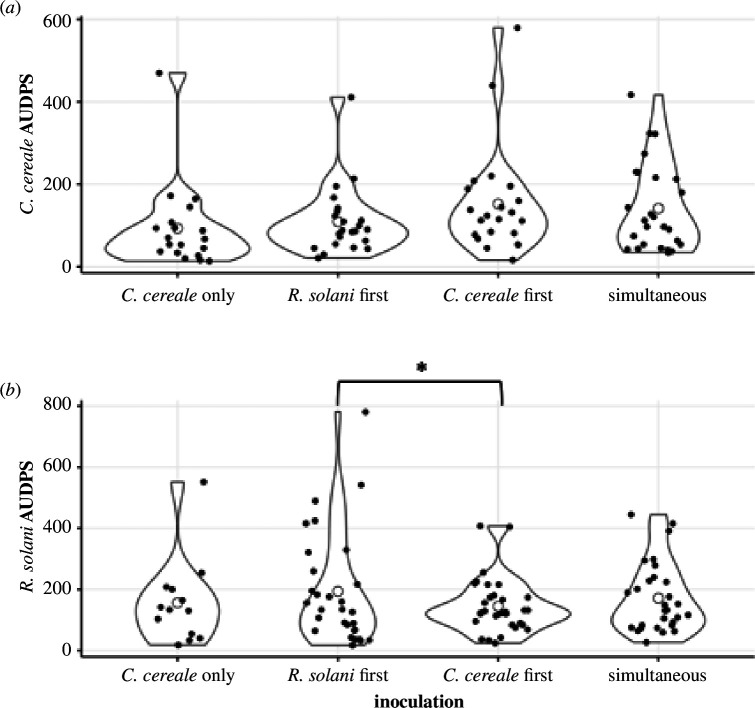
Distribution of area under the disease progress stairs (AUDPS) of *C. cereale* (*a*) and *R. solani* (*b*) lesion lengths for tall fescue plants with symptomatic infection. Open circles represent the inoculation group mean values. Significant differences in *R. solani* disease severity between inoculation groups are noted by asterisks.

Analysing the disease progression over time, we found that the severity of *R. solani* lesions in terms of AUDPS were marginally significantly affected by *C. cereale* inoculation (*F*_3,95_ = 2.52, *p* = 0.063; figure 2*a*; electronic supplementary material, table S4). When broken down into pairwise comparisons, plants inoculated with *C. cereale* second experienced 15% more severe *R. solani* lesions as measured by lesion length over time than those that were first inoculated with *C. cereale* (Tukey HSD, *p* = 0.033). We found no effect of fertilization on *R. solani* infection (nutrients: *F*_1,95_ = 0.01, *p* = 0.964; inoculation × nutrients: *F*_3,95_ = 0.88, *p* = 0.455; electronic supplementary material, table S4). The severity of *C. cereale* infection in terms of AUDPS was not significantly affected by inoculation group, or nutrient treatment (inoculation: *F*_3,80_ = 1.39, *p* = 0.251; nutrients: *F*_1,80_ = 0.24, *p* = 0.6253; inoculation × nutrients: *F*_3,80_ = 1.66, *p* = 0.181; figure 2*b*; electronic supplementary material, table S5).

### (c) Host growth and nutrient allocation

**Figure 3.**
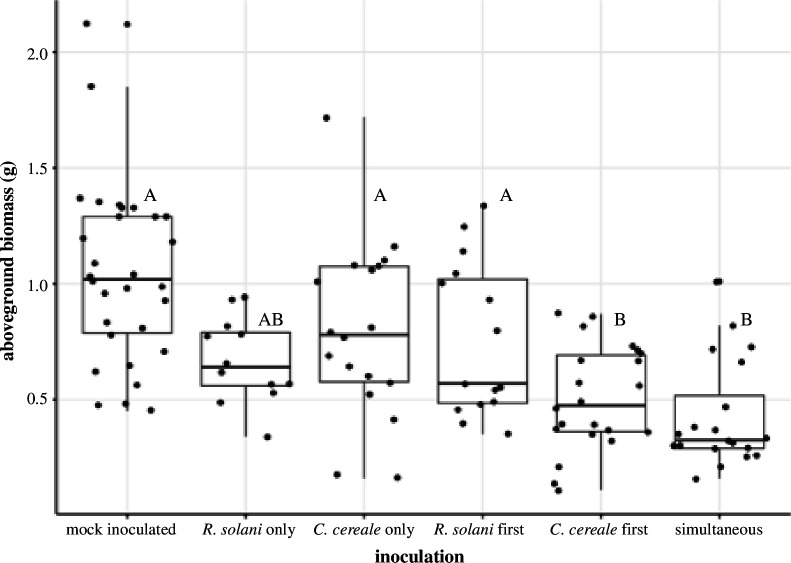
Total aboveground biomass of tall fescue plants by inoculation, with *R. solani* and/or *C. cereale*. Box illustrates the median and the first (lower boundary) and third (upper boundary) quartile of data with whiskers extending 1.5 times the interquartile range. Significant differences between inoculation groups are noted by letters.

Aboveground biomass differed between inoculation groups (*F*_5,105_ = 12.15, *p* < 0.001; figure 3; electronic supplementary material, table S6), but not between nutrient groups (nutrients: *F*_1,103_ = 0.83, *p* = 0.37; nutrients × inoculation: *F*_3,103_ = 0.30, *p* = 0.91; figure 3; electronic supplementary material, table S6). Plants inoculated with *C. cereale* first followed by *R. solani* or simultaneously with both pathogens had 16% and 24% reduced biomass respectively compared to the mock inoculated plants (Tukey HSD: *C. cereale* first, *p* = 0.0019: simultaneous, *p* < 0.001).

**Figure 4.**
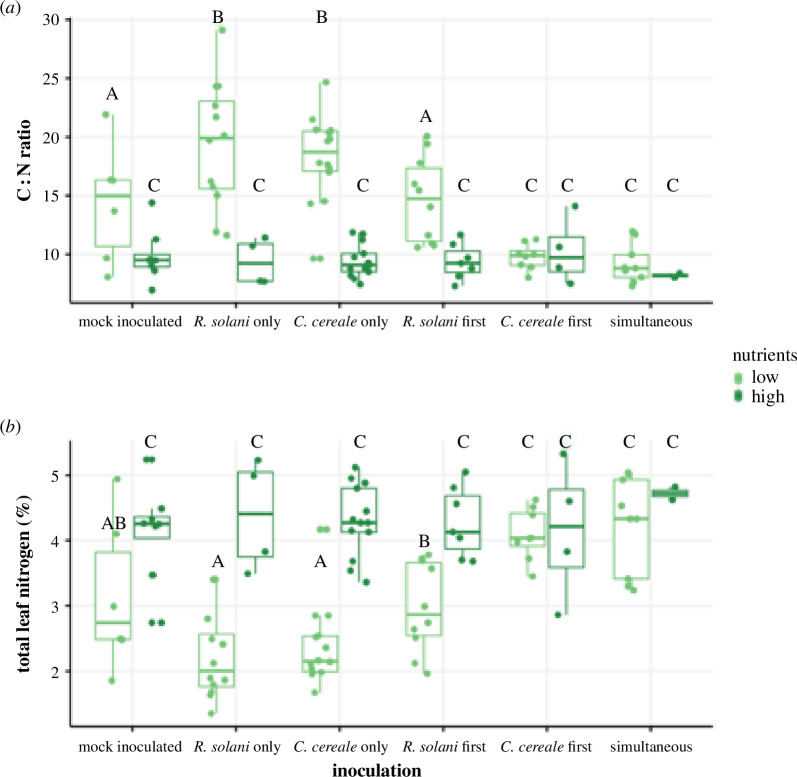
C : N (carbon-to-nitrogen) ratio (*a*) and total leaf nitrogen (%) (*b*) of the inoculated leaf of tall fescue by inoculation, with *R. solani* and/or *C. cereale*, and nutrient treatment, low (50 ppm N–P–K) and high (150 ppm N–P–K). Box illustrates median and the first (lower boundary) and third (upper boundary) quartile of data with whiskers extending 1.5 times the interquartile range. Significant differences between nutrient and inoculation group combinations are noted by letters.

Plants that received high nutrient addition had lower leaf C : N ratios compared to the low nutrient plants regardless of inoculation treatment (nutrients: *F*_1,86_ = 73.85, *p* = <0.001; figure 4*a*; electronic supplementary material, table S7). Of the low nutrient plants, those that were inoculated with only *R. solani* or *C. cereale* had the highest C : N ratio. Mock inoculated plants had 30% lower C : N ratio and plants inoculated with *R. solani* first had 33% lower C : N ratio relative to those inoculated with *R. solani* first (Tukey HSD: mock inoculated, *p* = 0.0306; *R. solani* first, *p* = 0.0044). Finally, low nutrient plants that were inoculated first with *C. cereale* or with both pathogens simultaneously had at least a 49% reduction in leaf C : N ratios (Tukey HSD: *C. cereale* first, *p* < 0.001; simultaneous, *p* < 0.001) and their C : N ratios were similar to all plants in the high nutrient group (inoculation: *F*_5,86_ = 11.16, *p* = <0.001; inoculation × nutrients: *F*_5,86_ = 5.47, *p* = <0.001; figure 4*a*; electronic supplementary material, table S7). Total leaf nitrogen followed similar trends, with the high fertilizer plants having increased leaf nitrogen (nutrients: *F*_1,83_ = 71.26, *p* < 0.001; figure 4*b*; electronic supplementary material, table S8) and the leaf nitrogen in low fertilizer plants increasing with simultaneous inoculation or *C. cereale* first inoculation (inoculation: *F*_5,83_ = 10.55, *p* < 0.001; inoculation × nutrients: *F*_5,83_ = 5.05, *p* < 0.001; figure 4*b*; electronic supplementary material, table S8).

### (d) Post hoc hypothesis

After controlling for the duration and severity of pathogen infection, inoculation treatment continued to explain a significant amount of the variation in aboveground biomass (*R. solani*: *F*_5,112_ = 3.26, *p* = 0.009; electronic supplementary material, table S9; *C. cereale*: *F*_5,112_ = 9.33, *p* < 0.001; electronic supplementary material, table S11), while the interaction between inoculation and nutrient addition remained a significant predictor of total leaf nitrogen (*R. solani*: *F*_5,94_ = 4.33, *p* = 0.0015; electronic supplementary material, table S10; *C. cereale*: *F*_5,94_ = 5.56, *p* < 0.001; electronic supplementary material, table S11). Aboveground biomass and total leaf nitrogen were partially explained by the duration and severity of infection. Aboveground biomass decreased as the duration of infection by *R. solani* increased (*F*_1,112_ = 43.81, *p* < 0.001; electronic supplementary material, table S9). Aboveground biomass also decreased as the duration of infection by *C. cereale* increased (*F*_1,112_ = 7.29, *p* = 0.0081; electronic supplementary material, table S11) and decreased as severity of infection increased (*F*_1,112_ = 5.95, *p* = 0.0165; electronic supplementary material, table S11). Only severity of *C. cereale* infection influenced leaf nitrogen, which increased with severity (*F*_1,94_ = 19.36, *p* = <0.001; electronic supplementary material, table S12).

## 4. Discussion

Contrary to our predictions and previous studies [32,33,53], nutrient addition did not influence infection incidence or severity. However, plants that were inoculated with *R. solani* alone experienced a decreased rate of infection compared to those inoculated with *C. cereale* either before, after or simultaneously. Additionally, plants inoculated with *C. cereale* first or simultaneously had reduced aboveground biomass, but increased leaf nitrogen concentrations when receiving the low nutrient addition treatment. When considered together, the results support facilitation of *R. solani* by *C. cereale* [11] and a growth-defence trade-off when the host plant is co-infected by pathogens of different trophic strategies [12,31]. Considering pathogen interactions in concert with host defence trade-offs may be key to understanding the outcomes of pathogen co-infections for host performance more broadly.

Inoculation with a second pathogen was the primary force driving infection rate, as previously found for disease severity by O’Keeffe *et al*. [11]. *Colletotrichum cereale* facilitated infection rates by *R. solani*, with *R. solani* having its lowest infection rate when infecting alone. This facilitation partially supports our first prediction that the order of arrival would affect infection success by way of the plant defence mechanisms, thereby modulating host susceptibility.

The facilitation may be explained by interactions between the induced plant defence hormones [4,13,54]. As a hemibiotroph, the primary defence hormone against infection by *C. cereale* is SA [12,55]. However, the primary defence hormone against necrotrophs like *R. solani* is JA [12]. When SA is induced, it can inhibit the JA pathway. This interaction has been shown experimentally in many systems [20,24]. There are at least 12 genes involved in negative crosstalk between the JA and SA pathways, which results in a plant only being able to upregulate one defensive hormone at a time [56]. By ramping up SA production in response to the arrival of *C. cereale*, the leaf may have been left susceptible to infection by the necrotophic *R. solani*. Further experiments to quantify SA and JA production and experimentally induce or block the defence pathways are necessary to test this mechanism as the source of facilitation between *C. cereale* and *R. solani*, but previous studies have documented the antagonistic interaction between SA and JA and confirmed its role in co-infection of multiple pathogens in monocots [55,57].

Neither pathogen was affected by nutrient availability. Pathogen infection rates and severity were the same between high and low nutrient plants and the facilitation of *R. solani* by *C. cereale* occurred regardless of nutrient treatment. The lack of interactive effects of nutrient and order of arrival on infection rate or severity suggest that this facilitation is not mediated by pathogen competition for nutrients within the leaves. We thus do not have support for our second prediction, that host nutrient availability would influence the severity of infection by mediating competition between co-infecting pathogens. The two focal pathogens occupy different trophic niches within the leaf environment as a necrotroph and a hemibiotroph, so this result indirectly lends support to the niche-based models of historical contingency [5,58]. Upon infection, *C. cereale* extracts nutrients from living plant tissue for a period before switching over to necrotrophy, while *R. solani* kills the plant tissue immediately to extract nutrients. While *C. cereale* is feeding as a biotroph it is not directly competing for the same host tissue resources as *R. solani* [8,12]. Another possible explanation for the lack of nutrient effect on disease progression is that the presence of the endophyte *E. coenophiala* conferred enough resistance or tolerance against infection that any potential effect of nutrients was overshadowed. Previous work has shown grass endophyte infection can increase host tolerance and resistance through production of defensive alkaloids and regulation of leaf senescence and photosynthetic capacity [41,42,44]. The severity of *R. solani* infection was greater in the second replicate, which could be owing to timing, location (growth room instead of growth chambers), or random chance.

Aboveground biomass mass was also not sensitive to nutrient treatment in the timeframe of our experiment. However, plants receiving the high nutrient treatment had reduced leaf C : N ratios and increased leaf nitrogen concentrations relative to those that received the low nutrient treatment. Together, these results are at least partially consistent with our third prediction. Nutrient availability did not affect plant growth in 5.5–6 weeks of fertilizer treatment, but it did influence leaf nitrogen concentration. If the plants had been allowed to continue growing, we may have seen aboveground biomass diverge between the nutrient treatments. Previous work has found leaf nitrogen to be one of the plant traits most sensitive to nutrient addition [59]. The difference in leaf nitrogen, but not plant biomass, between nutrient treatments supports the fact that leaf nitrogen is more sensitive to nutrient addition, especially given the short window of growth in this study. Additionally, infection with several species of endophytic *Epichloë* in different grass host species has been linked to increased host tolerance of abiotic stressors including nutrient limitation [45,60,61], suggesting that our plants’ infection with *E. coenophiala* could explain their lack of biomass response to nutrient addition, but, previous studies have found *E. coenophiala*-infected tall fescue growth to be sensitive to nutrients [10,15,36], so *E. coenophiala* cannot be assumed to be the only reason for the lack of effect of nutrients on growth in our study. Finally, leaf C : N and total leaf nitrogen, but not plant biomass, were greater in the first experimental replicate, perhaps because plants in the first replicate received one additional nutrient treatment as they had 5 days longer to grow.

In response to pathogen infection, plants may either divert nitrogen to the damaged leaves to fuel their defence or reduce growth of the damaged leaf and send carbon to the healthy portion of the plant [14,62]. Supporting our third prediction, co-inoculation of *C. cereale* and *R. solani*, either simultaneously or with *C. cereale* first, reduced plant growth and increased leaf nitrogen in the low fertilizer plants, resulting in nitrogen concentrations and C : N ratios that were indistinguishable from the high fertilizer plants. There is also documented crosstalk between induced defence hormones and growth hormones, suggesting that JA and SA production is at the expense of growth [56,63]. These two mechanisms, increasing leaf nitrogen and decreasing growth under co-infection relative to single infection, support a growth-defence trade-off. A host’s allocation to growth versus defence may be critically determined by whether it experiences a single pathogen infection or a co-infection [19,21,31,64].

One possible explanation for the reallocation of host resources when plants are inoculated with *C. cereale* first or both pathogens simultaneously is that the duration and intensity of infection alters plant resource allocation. If a pathogen infects early and severely, there is more time for it to extract nutrients from the host and alter plant growth and leaf nutrient concentrations [65,66]. In our post hoc analysis, we assessed the effect of duration and severity of infection for both *R. solani* and *C. cereale* on aboveground biomass and leaf nitrogen concentration. We found some support for this hypothesis. The severity of *C. cereale* infection was positively correlated with leaf nitrogen. The patterns were reversed for the effect of *C. cereale* on aboveground biomass: biomass was negatively correlated with the duration and severity of *C. cereale* infection.

The inconsistent effects of duration and severity of infection between leaf nitrogen and aboveground biomass further support the growth-defence hypothesis. When plants were infected for a longer duration or at a higher intensity, they had increased leaf nitrogen suggesting that the plant was reallocating nutrients to the highly infected leaf. Additionally, the analysis suggests that plants decreased growth as the duration and severity of *C. cereale* infection increased. These patterns add support to the inoculation treatment and order of pathogen arrival being important predictors of plant responses to infection.

When evaluated together, the results of this study suggest that host defences act as central linkages from host nutrient availability and order of pathogen arrival to infection outcomes for both pathogens and hosts. Previous studies have assessed the intersection between nutrient addition and infection, finding a general increase in disease severity of obligate pathogens and a decrease in severity of facultative pathogens [29]. Additionally, research has found priority effects to alter infection rates and severity of disease in co-infection [8,9,13] and to be important in host fitness and response to infection [7,67]. By integrating both infection rate and severity with host nutrient concentrations our study outlines a pathway for future studies to tease out how nutrient availability interacts with plant defences against multiple pathogens whose interactions are subject to priority effects.

## Data Availability

The data that support the findings of this study are openly available on Dryad [68]. Supplementary material is available online [69].
